# In-plane optical anisotropy of InAs/GaSb superlattices with alternate interfaces

**DOI:** 10.1186/1556-276X-8-298

**Published:** 2013-06-25

**Authors:** Shujie Wu, Yonghai Chen, Jinling Yu, Hansong Gao, Chongyun Jiang, Huang, Yanhua Zhang, Yang Wei, Wenquan Ma

**Affiliations:** 1 Key Laboratory of Semiconductor Materials Science, Institute of Semiconductors, Chinese Academy of Sciences, P.O. Box 912, Beijing 100083, People’s Republic of China; 2 Beijing Key Laboratory of Low Dimensional Semiconductor Materials and Devices, Institute of Semiconductors, Chinese Academy of Sciences, P.O. Box 912, Beijing 100083, People’s Republic of China; 3 Laboratory of Nano-Optoelectronics, Institute of Semiconductors, Chinese Academy of Sciences, P.O. Box 912, Beijing 100083, People’s Republic of China

**Keywords:** In-plane optical anisotropy, InAs/GaSb superlattices, Reflectance difference spectroscopy, 78.67.Pt, 78.66.Fd, 78.40.Fy

## Abstract

The in-plane optical anisotropy (IPOA) in InAs/GaSb superlattices has been studied by reflectance difference spectroscopy (RDS) at different temperatures ranging from 80 to 300 K. We introduce alternate GaAs- and InSb-like interfaces (IFs), which cause the symmetry reduced from *D*_2*d*_ to *C*_2*v*_. IPOA has been observed in the (001) plane along [110] and [1

$$\bar{1}$$

0] axes. RDS measurement results show strong anisotropy resonance near critical point (CP) energies of InAs and GaSb. The energy positions show red shift and RDS intensity decreases with the increasing temperature. For the superlattice sample with the thicker InSb-like IFs, energy positions show red shift, and the spectra exhibit stronger IPOA. The excitonic effect is clearly observed by RDS at low temperatures. It demonstrates that biaxial strain results in the shift of the CP energies and IPOA is enhanced by the further localization of the carriers in InSb-like IFs.

## Background

**Figure 1:**
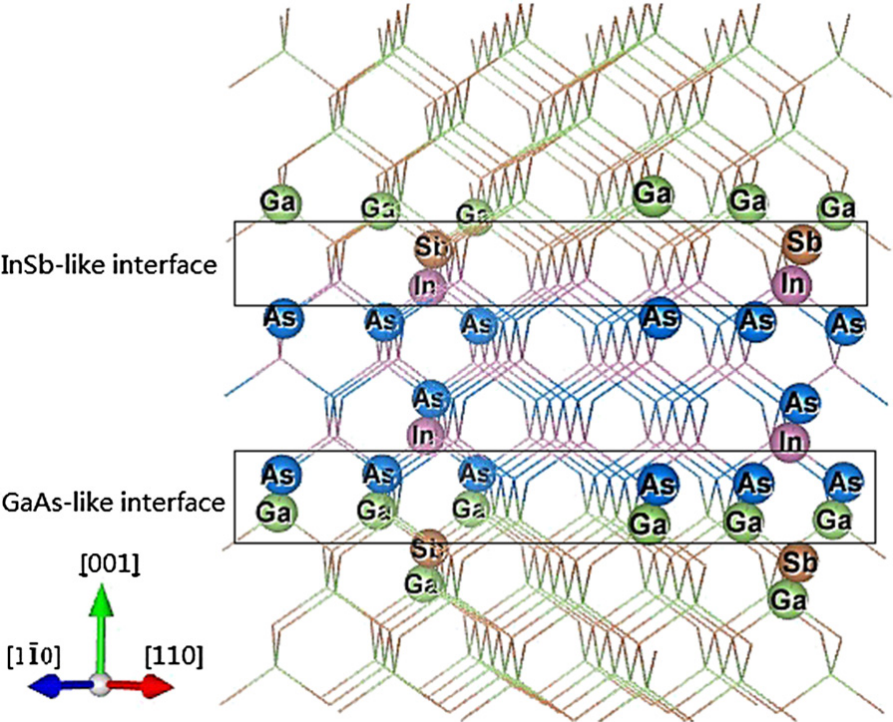
**Simple stick-and-ball model of InAs/GaSb SL with alternate GaAs and InSb IFs.** The purple, blue, green, and brown balls denote In, As, Ga, and Sb atoms, respectively.

InAs/GaSb type-II superlattices (SLs) are a considerable interest in the application of middle and far infrared photodetection. These structures have broken-gap band alignment, which allows tuning optical and electronic properties by varying layer thickness [1,2]. As the InAs and GaSb share no common atoms (NCA) across the interface (IF), these IFs have to be controlled by both InAs-like, both GaSb-like or alternating InAs- and GaSb-like. Figure 1 illustrates a simplified ball-and-stick model of InAs/GaSb SL with lower GaAs-like and upper InSb-like IFs. This kind of CA/C’A’ zinc blende hetero-structures lost their ideal *T*_*d*_ point-group symmetry along the [001] growth direction. C and A represent cation and anion, respectively. If SLs have only one type of IF such as C-A’ or C’-A, it exists a *S*_4_ rotation-reflection axis, the symmetry is described as *D*_2*d*_ point-group symmetry. If SLs have both kinds of IFs alternately, the symmetry depends on the number of atomic monolayer (ML) of each components. SLs components with one or both odd numbers of atomic ML belong to the *D*_2*d*_ point-group symmetry, with both even numbers of atomic ML are corresponding to *C*_2*v*_ point-group symmetry. If the structure shares a common atom (CA) (A=A’ or C=C’), the IFs have a *S*_4_ rotation-reflection axis corresponding to the *D*_2*d*_ point-group symmetry. It is supposed that C-A bonds lie in the (110) plane and A-C’ bonds are in the (1$\bar{1}$0) plane. When a beam of linear polarized light propagates along the [001] direction with its polarized direction parallel to the [110] or [1$\bar{1}$0] direction, it feels different chemical bonds. This kind of anisotropic-chemical-bond arrangement leads to in-plane optical anisotropy (IPOA) at the IFs, i.e., optical property of [110] and [1$\bar{1}$0] plane is different in the (001) plane. Exactly speaking, the IPOA of upper and lower IFs will cancel each other for the SLs with *D*_2*d*_ symmetry. Although, it is hard to realize such perfect IFs by the growth process that has many uncontrollable factors, the weak IPOA is still well observed by reflectance difference spectroscopy (RDS) [3,4]. For the NCA SLs, it has been observed that the IPOA is very strong [5-8].

RDS is a very sensitive nondestructive optical detection technique for IPOA, which was invented by D.E Aspens [9]. This powerful tool is used to detect IPOA induced by strain, electric field, and atom segregation for bulk, surface, and IF. In this letter, we have measured the IPOA of (001) plane of InAs/GaSb SLs by RDS at different temperatures ranging from 80 to 300 K. In this experiment, two SL examples have different thickness of InSb-like IF. The spectra are ranging from 1.5 to 5.0 eV. In the spectra, the energies of main features are assigned to Γ (*E*_0_, *E*_0_+*Δ*_0_), Λ (*E*_1_, *E*_1_+*Δ*_1_), and other critical point (CP) interband transitions of InAs, GaSb, and the coupling of the components whereas the *L*, *X*, and *Σ* CP energies are complex and difficult to analyze. Table 1 shows a list of the CP energies of bulk InAs, GaSb, GaAs, and InSb [10]. Additional CP energies may be related to the IFs. The Λ CP energies are very sensitive to strain. The CP energies show red shift with the increasing temperature, which attributes to the enhancement of electron-phonon interaction and thermal expansion. The transitions show a clear exciton characteristic at low temperatures. Compared with sample A, the measured energies of Λ CPs show red shift for sample B and exhibit stronger IPOA. The red shift attributes to the increasing of average lattice constant. IPOA is enhanced by the further localization of carriers in InSb-like IFs.

**Table 1:** **CP energies (in eV) of bulk InAs, GaSb, GaAs, and InSb measured by S.Adachi [**[10]**]**

	**InAs**	**GaSb**	**InSb**	**GaAs**
*E*_0_	0.36	0.72	0.18	1.42
*E*_0_ + Δ _0_	0.76	1.46	0.99	1.77
*E*_1_	2.50	2.05	1.80	2.90
*E*_1_+ Δ _1_	2.78	2.50	2.30	3.13
*E*_2_	4.45	4.00	3.90	4.70

## Methods

The InAs/GaSb SLs were grown on GaSb buffer layer, which is deposited on non-intentional doping GaSb (001) substrates by molecular beam epitaxy (MBE). The GaAs-like IFs were generated by employing As soaking after GaSb is deposited. The InSb-like IFs were formed by InSb deposition. Two samples have the same structure as 100 periods InAs (10 ML)/GaSb (8 ML) without capping layer. The difference of the two examples is only the thickness of InSb layer, 0.43 ML (sample A) and 1.29 ML (sample B), respectively. We used a Bede D1 high-resolution X-ray diffractometer to characterize structural quality of the samples. The lattice mismatch and one-period thickness can be predicted.

We measured the relative reflectance difference between [110] and [1$\bar{1}$0] in (001) plane, obtaining

(1) $$\begin{array}{lcrr} \frac{\mathrm{\Delta r}}{r}=\frac{r_{110}-r_{1\bar{1}0}}{r_{110}+r_{1\bar{1}0}}, & & & \end{array}$$

ranging from 80 to 300 K in a cryogenic Dewar bottle. In the RDS measurement, near-normal incidence reflectivity of two perpendicular directions was obtained in order to remove the influence of errors induced by optical components, averaging two spectra sample azimuth by 90°. The difference of dielectric functions ($\Delta \varepsilon =\varepsilon_{110}-\varepsilon_{1\bar{1}0}$) has a relation with Δr/r:

(2) $$\begin{array}{lcrr} \;\frac{\mathrm{\Delta r}}{r}\;=\;(\alpha \;+\;\mathrm{i\beta})\Delta \varepsilon \;=\;(\alpha \Delta \varepsilon_{r}\;-\;\beta \Delta \varepsilon_{i})\;+\;i(\alpha \Delta \varepsilon_{i}\;+\;\beta \Delta \varepsilon_{r}). & & & \end{array}$$

Here, *α* and *β* are complicated functions of four refractive indices and the wavelength of light. Both the real and imaginary part of *Δ*r/r are linear combinations of real and imaginary part of *Δε*[11]. The degree of polarization (DOP) is defined as $P=\frac{M_{110}-M_{1\bar{1}0}}{M_{110}+M_{1\bar{1}0}}$ (*M*_110_ is the transition probability when light is polarized along [110] direction). Im(*Δε*) is proportional to *ΔM*, and Im(*ε*) is proportional to *M*. It can be deduced from the imaginary part of *Δε* and the imaginary part of *ε*: $\left(P=\frac{\text{Im}(\Delta \varepsilon )}{2\text{Im}(\varepsilon )}\right)$[12].

## Results and discussion

Lattice constants of GaAs, InAs, GaSb, and InSb are 5.2430, 6.0173, 6.0959, and 6.8970 Å, respectively [13]. The lattice mismatch between InAs and GaSb is only 0.6%; however, that of GaAs/GaSb and InSb/GaSb are 8% and 6%, respectively. Inserting GaAs-like IFs equals to introduce compress strain for the SLs, while InSb-like IFs will result in tensile strain. Alternating GaAs- or InSb-like IF layers can compensate the lattice mismatch between InAs and GaSb by controlling the appropriate thickness of GaAs and InSb layers. If SLs are pseudomorphic-grown on GaSb substrate, the strains of GaAs, InAs, and InSb are determined by the substrate, which can be calculated by:

(3) $$\begin{array}{lcrr} e_{z}^{i}=\frac{a_{⊥}^{i}-a^{i}}{a^{i}},e_{x}^{i}=e_{y}^{i}=\frac{2v_{i}}{1-v_{i}}e_{z}^{i}. & & & \end{array}$$

$e_{z}^{i}$, $e_{x}^{i}$, and $e_{y}^{i}$ are the strains of GaAs, InAs, and GaSb for directions parallel and perpendicular to the growth direction, respectively. *a*_*sub*_, *a*_*i*_, and $a_{⊥}^{i}$ represent crystal constants of GaSb substrate, for each layer, and the layers of SLs after growth, respectively. *v*_*i*_ is the Possion ratio. The band gap and energies of CPs will show blue or red shift for compress or tensile biaxial strain, respectively. The two SL samples have the same thickness of GaAs-like IFs and different thickness of InSb-like IFs. The average lattice constant of superlattice is increased as a result of red shift energies of the CPs.

X-ray diffraction (XRD) results indicate that the range of 0th peak of sample A and the substrate is 0.367° and 0.151° for sample B. The full width at half maximum (FWHM) of the first satellite peak is 34 arcsec for sample A and 43 aresec for sample B. Both of the samples show compression strain. The calculated strain is -0.0054 for sample A and -0.0023 for sample B. Increasing the thickness of InSb-like IF layers can reduce the average compression strain. We predicted one-period thickness from the spacing between the satellites. Each period thickness of sample A is 55.9 Å and 56.8 Å for sample B.

**Figure 2:**
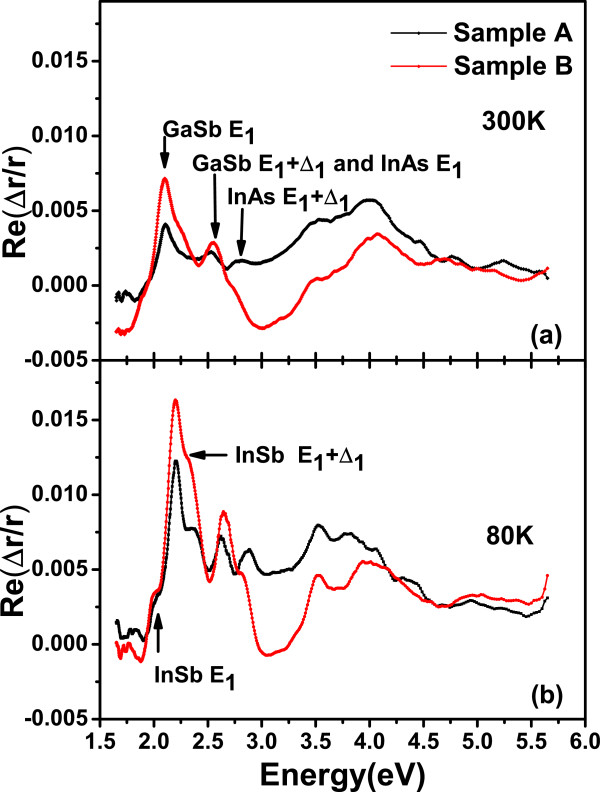
**Real part of RD spectra of samples A and B measured at 300 and 80 K.** (**a**) At 300 K. (**b**) At 80 K. The arrows indicate the CP energies.

Figure 2a,b shows the real parts of the relative reflectance difference measured at 300 and 80 K, respectively. The resonances of two samples have the same lineshape. In the spectra, the sharp peak near 2.05 eV(CP1), which is related to *E*_1_energy of GaSb. The lineshape of real part is almost the derivative of the imaginary part. A small feature is observed at this region, which is coincidence that the InAs *E*_1_ and GaSb *E*_1_+*Δ*_1_energies are both near 2.50 eV(CP2). The InAs *E*_1_energy is a little larger than GaSb *E*_1_+*Δ*_1_ energy. Another feature is observed near 2.78 eV(CP3) corresponding to the critical point energy of InAs *E*_1_+*Δ*_1_. Two shoulder-like features were marked in Figure 2b on both sides of the sharp peak near 2.05 eV, which may be attributed to InSb-like IFs. The energy positions are near the *E*_1_ and *E*_1_+*Δ*_1_energies of bulk InSb, and it is more clearly shown in the 80-K measurement. However, the IPOA structures about GaAs are not observed. In comparison with sample A, it is observed that GaSb *E*_1_ and InAs *E*_1_+*Δ*_1_features show red shift for sample B, which attributes to the compensation of stress by increasing the thickness of InSb-like IF layer. It is anomalous that a blue shift peak is corresponding to InAs *E*_1_ and GaSb *E*_1_+*Δ*_1_. D. Behr et al. reported that it is complicated by inhomogeneity for *E*_1_ and transition of InAs and *E*_1_+*Δ*_1_ of GaSb [14].

For SL sample, reflectivity can be described by a three-phase model:

(4) $$\begin{array}{lcrr} r=\frac{V_{23}+V_{12}\exp(-i2\varphi_{2})}{1+V_{23}V_{12}\exp(-i2\varphi_{2})}; & & & \end{array}$$

with

() $$\begin{array}{lcrr} V_{\text{ij}}=\frac{{\tilde{n}}_{j}-{\tilde{n}}_{i}}{{\tilde{n}}_{j}-{\tilde{n}}_{i}},\varphi_{2}=\frac{2\pi {\tilde{n}}_{2}d_{2}}{\lambda }, & & & \end{array}$$

where the indices *i* and *j* take the value 1, 2, and 3 for the substrate, SL layer, and air, respectively. ${\tilde{n}}_{i}$ is complex refractive index of the *i*th layer, *d*_2_is the thickness of the SL layer, Λ is the wavelength of light in vacuum [15]. SL layer are treated as uniaxial medium, ${\tilde{n}}_{2}$ is the weighted average refractive index of 100 periods of InAs (10 ML)/GaSb (8 ML) SL layer. We chose a simple three-phase model, with no capping layer:

(5) $$\begin{array}{lcrr} \frac{\mathrm{\Delta r}}{r}=-\frac{4\mathrm{\pi id\Delta \varepsilon}}{\lambda (\varepsilon_{s}-1)}. & & & \end{array}$$

**Figure 3:**
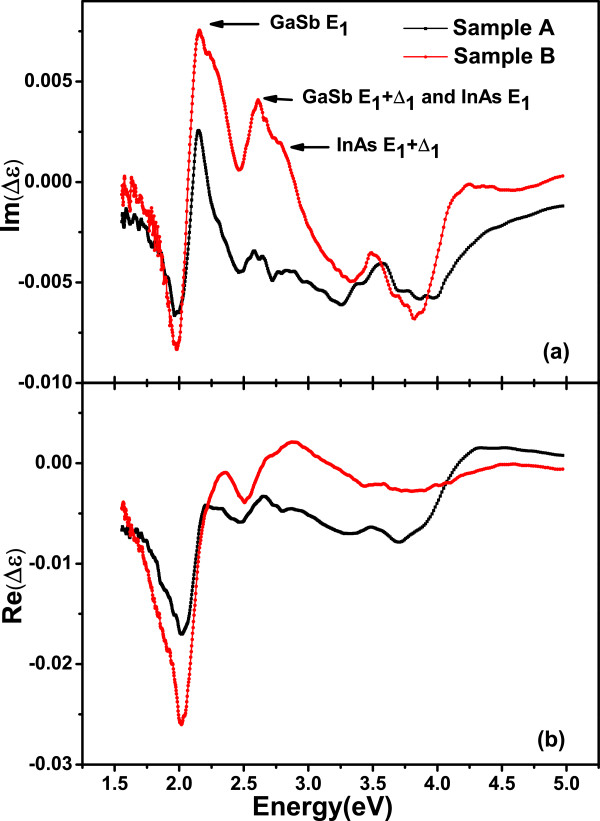
**Calculated imaginary (a) and real (b) parts of Δ *ε* of samples A and B.** The arrows indicate the CP energies.

*ε*_*s*_ is the dielectric function of GaSb substrate, *d* is the thickness of the superlattice, and Λ is the wavelength of light [16]. The *ε*_*s*_data of GaSb substrate is taken from Aspnes’ measurement [17]. Figure 3a,b shows the real and imaginary parts of anisotropy dielectric function *Δε* by Equation 5, respectively. The peaks and valleys in the imaginary anisotropic dielectric function spectra are corresponding to the CP energies. The imaginary part of *ε* is related to absorption, and the real part corresponds to transmittance properties.

**Figure 4:**
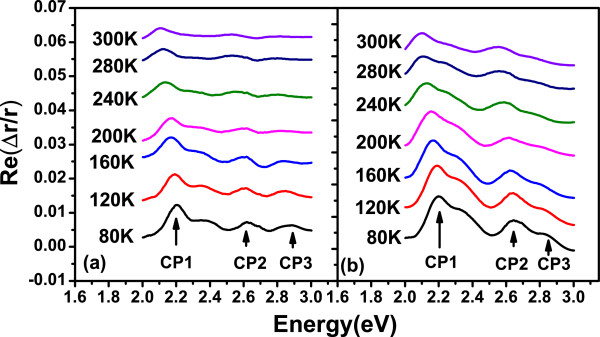
**Real part ofΔ r/r of samples A and B measured ranging from 80 to 300 K.**

**Figure 5:**
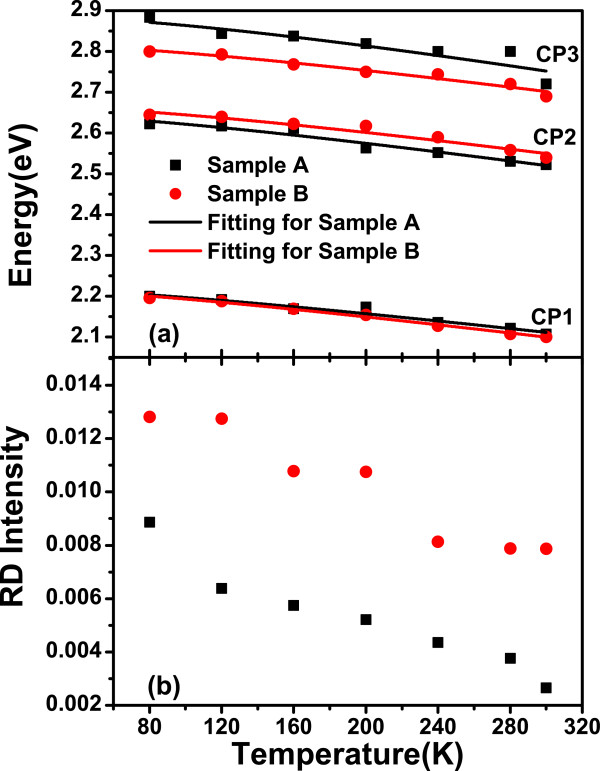
**Measured CP energies of samples A and B as function of temperature and RD instensity of CP1.** (**a**) Measured CP energies of samples A (squares) and B (circles) as a function of temperature. The lines are the Varshni empirical formula fitting. (**b**) Temperature-dependent RD intensity of CP1.

Figure 4a,b shows the measured IPOA of samples at different temperatures ranging from 80 to 300K. Figure 5a shows the temperature dependence of measured CP energy positions. Figure 5b shows the reflectance difference intensity of CP1 as a function of temperature. The energies of CPs show blue shift, and the amplitudes increase with the decreasing of measured temperature. There are no additional peaks observed. All the observed features are corresponding to CP energies. This kind of IPOA is stable and not caused by defects accumulated on the IF. The shoulder-like CP energy features about InSb clearly show character at low temperatures. Compared with sample A, all the spectra measured at different temperatures indicate that the CP energy are positioned on the red shift with a stronger RD intensity for sample B. J.S. Hang has reported that the GaSb critical point energies shift with temperature, as described by the Varshni expression [18], while J. Kim described the InAs CP energies and temperature dependence as Bose-Einstein statics [19]. We use the Varshni empirical formula to fit the temperature dependence:

(6) $$\begin{array}{lcrr} E(T)=E_{0}-\frac{\alpha T^{2}}{T+\beta }, & & & \end{array}$$

where *β* is a constant (K), *E*_*o*_is the width of semiconductor band gap, *α* is a fitting parameter (eVK^−1^), and *T* is the temperature. Table 2 lists the Varshni coefficients of samples A and B. It is found that excitonic transitions have important contributions to *E*_1_ and *E*_1_+*Δ*_1_ transitions. For this kind of transitions along eight equivalent Λ axes 〈111〉 direction of the Brillouin zone, the FWHM of the spectra decreases with the temperature decreasing. Since the spin orbit interaction in the valence band is large, the *E*_1_ transition split into *E*_**1**_and *E*_1_+*Δ*_1_ transitions. *Δ*_1_ is approximately 2/3 of Δ _0_ at the Brillouin zone center [20]. The symmetry reduction remove the degeneracy of the four equivalent bands of two sets. As mentioned above, *Δ*r/r is related to *Δε*; therefore, the line shape also depends on the symmetry of CP [21]. One electron approximation cannot explain the lifetime broadening; thus, it is suggested that Coulomb interaction should be taken into consideration [22]. The sharpening of spectra with reduction temperature indicates that excitons associate with the *E*_1_ transition [23].

**Table 2:** Varshni parameters for temperature-dependence fitting CPs of samples A and B

**Sample**	**CPs**	***E***_***0***_**(eV)**	***α* 10 **^**−4**^**(*eVK***^**−1**^**)**	***β* (K)**
A	CP1	2.218	5.34	149
	CP2	2.646	6.45	160
	CP3	2.888	8.08	235
B	CP1	2.217	5.62	130
	CP2	2.666	6.44	198
	CP3	2.817	6.51	207

**Figure 6:**
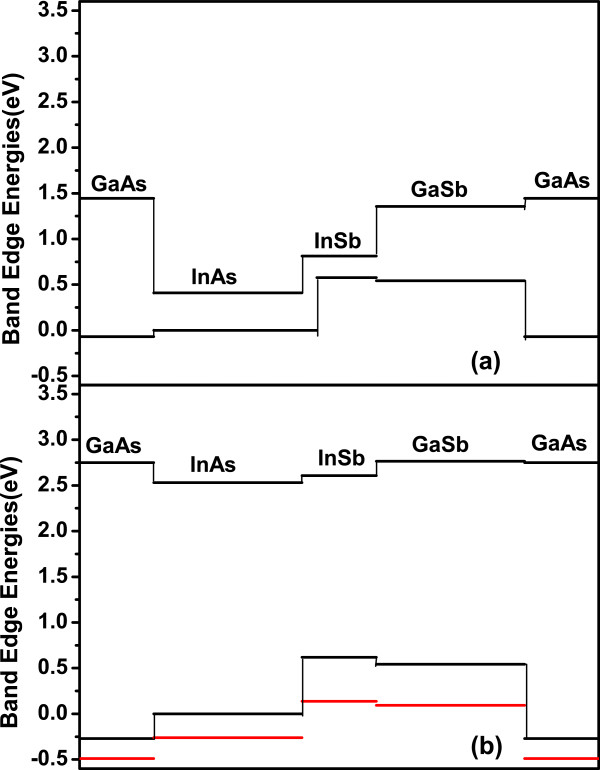
**Band alignments of InAs, GaAs, GaSb and InSb binary system.** (**a**) At Γ point of Brillouin zone. (**b**) At *L* point of Brillouin zone. The red lines are the spin-orbital splitting energies at *L* point.

Samples A and B are both with GaAs-like and InSb-like alternate IFs and even number of InAs and GaSb MLs. The SLs possess *C*_2*v*_symmetry in the ideal condition. At successive IFs, if In-Sb bonds lie in the (110) plane, while In-As bonds lie in the (1$\bar{1}$0) plane. Linearly polarized light propagates along the (001) direction. When the polarized direction is parallel to [110] and [1$\bar{1}$0] directions, it feels different chemical bonds at IFs. As a result, the optical properties along the [110] and [1$\bar{1}$0] directions are different. In the RDS spectra, InSb features were not observed clearly in room temperature, since the features of *E*_0_, *E*_1_, and *E*_1_+*Δ*_1_CPs are very broadening with few ML [24]. This effect is identified as the spread of carrier wave function of the ultra-thin IF to surrounding layers. Figure 6a shows the *ΔE*_*c*_and *ΔE*_*v*_ of unstrained GaAs, InAs, InSb, and GaSb system at *Γ* point [25,26]. *E*_1_and *E*_1_+*Δ*_1_take place along the Λ directions of the Brillouin zone where the valence and conduction bands are nearly parallel. The energy gap of *L* and Λ are nearly equal. We have inferred the band alignment of *L* point in Figure 6b. The reflectance peaks of *L* transitions are not observed, since these transitions are too weak or hidden in the Λ transition structures [22]. In Figure 6b, the Λ _1_conduction band offset between InAs and GaSb is 0.234 eV, and the Λ _3_valence band offset is 0.544 eV. The staggered band alignment of bulk materials imply that in every InAs/GaSb SL, there is a InAs-like conduction band minimum and GaSb-like valence band maximum. The Λ _3_valence band of InSb is much higher than GaSb, and the Λ _1_conduction band is much higher than InAs. The Λ _3_valence band splits into Λ _(4,5)_and Λ _6_since the spin-orbital interaction. The red lines show the Λ _6_energy positions. The Λ _6_band of InSb is higher than Λ _(4,5)_band of InAs. As the thickness of InSb layers is increasing from 0.43 to 1.29 ML, compared to sample A, the effect of quantum well structures is enhanced. More holes are localized in InSb layers. However, there is no such effect for the GaAs layer. The IPOA intensities of CP1, CP2, and the shoulder-like CP about InSb are increased. While the IPOA intensities of CP3 are decreased and the transition energy position of CP2 are anomalous, blue shift may attribute to the coupling of these states.

## Conclusions

The IPOA of InAs/GaSb SLs with InAs-like and GaSb-like alternate IFs were observed by RDS. The main mechanism can attribute to the symmetry reduction to *C*_2*v*_. The increasing of InSb IFs’ thickness release the mismatch between the SL layer and substrate. The red shift of CP energies was observed. Meanwhile, the holes are further localized in the InSb IFs, leading to the intensities of IPOA further increased.

## Abbreviations

IPOA: In-plane optical anisotropy; SLs: Superlattices; RDS: Reflectance difference spectroscopy; CP: Critical point; IFs: Interfaces; NCA: No common atom; ML: Monolayer; MBE: Molecular beam epitaxy; DOP: Degree of polarization.

## Competing interests

The authors declare that they have no competing interests.

## Authors’ contributions

SW carried out the analysis, did the measurements, and drafted the manuscript. YC conceived of the study and participated in its design and coordination. JY and HG participated in the design of the study. JY and CJ participated in the revision of the manuscript and discussed the analysis. JH, YZ, and YW prepared the samples and measured the quality by XRD. WM designed the structure and supervised the preparation of samples. All authors read and approved the final manuscript.
